# HIF-1*α* Is Associated with Resistance to Hypoxia-Induced Apoptosis in Ameloblastoma

**DOI:** 10.1155/2021/3060375

**Published:** 2021-12-27

**Authors:** Katherine Julissa Palma Valladares, Karolyny Martins Balbinot, Antonia Taiane Lopes de Moraes, Maria Sueli da Silva Kataoka, Aline Maria Pereira Cruz Ramos, Rommel Thiago Jucá Ramos, Artur Luiz da Costa da Silva, Ricardo Alves Mesquita, David Normando, Sérgio de Melo Alves Júnior, João de Jesus Viana Pinheiro

**Affiliations:** ^1^Laboratory of Pathological Anatomy and Immunohistochemistry, School of Dentistry, Federal University of Pará, Belém, PA, Brazil; ^2^Cell Culture Laboratory, School of Dentistry, Federal University of Pará, Belém, PA, Brazil; ^3^Health Science Institute, Federal University of Pará, Faculty of Nursing, Belém, PA, Brazil; ^4^Biological Engineer Laboratory, Park of Science and Technology, Belém, PA, Brazil; ^5^Department of Oral Surgery and Pathology, School of Dentistry, Federal University of Minas Gerais, Belo Horizonte, MG, Brazil; ^6^Department of Orthodontics, Federal University of Pará, Faculty of Dentistry, Belém, PA, Brazil

## Abstract

**Background:**

Ameloblastoma (AMB) is a benign odontogenic tumour, with an aggressive local behaviour and a high rate of recurrence. Previous studies have demonstrated that hypoxia-induced factor alpha 1 (HIF-1*α*) and activated caspase-3 contribute to tumour invasiveness and cytogenesis in ameloblastoma. Hypoxia increases HIF-1*α* levels, which triggers a number of signalling pathways. This paper aimed to present data in the study of hypoxia-activated signalling pathways that modulate proapoptotic and antiapoptotic events in AMB.

**Methods:**

Twenty cases of AMB and ten cases of dental follicle (DF) were used to analyse the immunoexpression of HIF-1*α*, p53, BNIP3, Bcl-2, IAP-2, GLUT1, and Bax. To contribute to the study, an analysis of expression and genetic interaction was performed using the cell line AME-1.

**Results:**

AMB and DF expressed the studied proteins. These proteins showed significantly greater immunoexpression in AMB compared with the DF (*p* < 0.05). HIF-1*α* showed an important association with GLUT1, and a positive correlation was observed among p53, Bcl-2, and IAP-2. Transcriptomic analysis showed the significant expression of the studied proteins, and the network generated showed a direct association of HIF-1*α*F with GLUT1 (SLC2A1), TP53, and LDHA. Interestingly, GLUT1 also exhibited direct interaction with TP53 and LDHA.

**Conclusion:**

In AMB tumorigenesis, hypoxia is possibly related to antiapoptotic events, which suggests an important role for HIF-1*α*, GLUT1, Bcl-2, IAP-2, and possibly p53.

## 1. Introduction

Ameloblastoma (AMB) is a locally invasive benign neoplasm. It represents between 10% and 45.2% of odontogenic tumours and approximately 1% of all neoplasms of the oral and maxillofacial region [1]. This tumour grows slowly and mainly affects the mandible, generally presenting an aggressive behaviour and may present with local recurrence [2]. Recurrence can reach 90% after conservative treatments, such as curettage and/or enucleation [1]. Thus, the treatment of choice is surgical removal, which can affect the oral and maxillofacial region both aesthetically and functionally [2].

In the tumorigenesis of AMB, several studies have tried to relate key proteins, signalling pathways, and some phenomena such as apoptosis [3–7]. Apoptosis is a cellular phenomenon that can be activated by hypoxia, and this is characteristic of most solid tumours. The hypoxic environment mainly results from oxygen consumption due to the rapid proliferation of tumour cells [4]. In the hypoxia, cells undergo a variety of adaptive responses that include activation of signalling pathways, which promotes cell survival or death. HIF-1*α* is the main transcription factor that mediates these adaptive responses [5].

HIF-1*α* exerts its antiapoptotic function through transcriptional activation of antiapoptotic proteins: Bcl-2, apoptosis inhibitor (IAP-2), and GLUT1. On the other hand, a functional pathway of HIF-1*α* can activate proapoptotic proteins: p53, Bax, and BNIP3 [5].

Considering the previous studies, our research group observed that HIF-1*α* is expressed in the nucleus of neoplastic cells in solid areas of the ameloblastoma, whereas it was expressed in the cytoplasm and nucleus in cystic regions [8, 9]. These different expressions in different areas could possibly induce different phenomena such as invasion in solid areas, and in other regions, apoptosis cold lead to cystic cavity formation.

Under normoxic conditions, the HIF-1*α* subunit is expressed but rapidly degraded. During hypoxia, the alpha subunit stabilises and translocates to the nucleus [10]. This causes its accumulation to be heterodimerised with the beta subunit (HIF-*β*), where it will bind to specific DNA sequences, activating genes involved in adapting to hypoxia, cell survival, angiogenesis, and metastasis [11, 12].

Therefore, considering the knowledge about tumorigenesis of AMB and proteins related to the hypoxia-induced apoptosis, the aim of this study is to deepen knowledge about hypoxia-activated signalling pathways, which modulate pro- and antiapoptotic events, through the study of HIF-1*α*, p53, BNIP3, Bcl-2, IAP-2, GLUT1, and Bax proteins in order to demonstrate data about the mechanism of these proteins in the tumorigenesis of AMB.

## 2. Materials and Methods

### 2.1. Samples

Tissue microarrays (TMA) with double core, 1.5 mm size, containing 20 ameloblastoma samples (AMBs) (TMA MC804, US Biomax Inc., Rockville, MD, USA) were used. All cores of TMA had parenchyma and stroma of AME. Neves-Silva et al. validated the use of TMA with ameloblastoma samples for studies with immunohistochemistry [13]. In addition, ten samples of paraffinic tissue from the dental follicle (DF) were retrieved from the archives of the Department of Maxillofacial Pathology, Faculty of Dentistry, Federal University of Pará (UFPA), Belém, PA, Brazil. 3 *µ*m sections of DF samples were mounted on loaded slides (Jiangsu Hiunda Medical Instruments Co., Ltd, Yancheng, JI, China). The samples were submitted to immunohistochemistry to detect the immunoexpression of HIF-1*α*, p53, BNIP3, Bcl-2, IAP-2, GLUT1, and Bax proteins. This study was approved by the Ethics Committee for Research with Human Beings of the Institute of Health Sciences, Federal University of Pará (nº 3.236.721). All methods were performed according to the Declaration of Helsinki.

### 2.2. Immunohistochemistry

The sections of the AMB and DF samples were deparaffinised in xylol and hydrated in decreasing concentrations of ethanol. The slides were immersed in a 3% hydrogen peroxide and methanol (1 : 1) solution for 30 min to inhibit endogenous peroxidase activity. Antigen retrieval was performed (30 sec, temperature of 120–125°C) in citrate buffer (pH 6.0) using a Pascal chamber (Dako, Carpinteria, CA, USA). Nonspecific antibody binding sites were blocked with 1% bovine serum albumin (BSA Sigma®) in phosphate buffered saline solution for one hour. The slides were incubated with the primary antibodies HIF-1*α* (1 : 50, clone HI*α*67, Millipore, Temecula, CA, USA), p53 (1 : 50, clone DO-1, Santa Cruz Biotechnology, Dallas, TX, USA), BNIP3 (1 : 25, clone Ana40, Santa Cruz®), Bcl-2 (1 : 25, clone C-2, Santa Cruz®), Bax (1 : 50, clone B-9, Santa Cruz®), GLUT1 (1 : 500, clone A-4, Santa Cruz®), and IAP-2 (1 : 200, clone DF6167, Affinity Biosciences, Cincinnati, OH, USA) for one hour. Subsequently, the sections were incubated with the secondary antibody Reveal (Spring Bioscience, Pleasanton, CA, USA) according to the manufacturer's instructions. Diaminobenzidine (Spring Bioscience®) was used as a chromogen. Mayer's haematoxylin (Sigma, San Luis, MO, USA) was used for counter staining and mounted using Permount^®^ (Fisher Scientific, Fair Lawn, NJ, USA). Samples of intraductal breast carcinoma were used as a positive control. The negative control was obtained by omitting the primary antibody, which was replaced by nonimmune serum.

### 2.3. Immunostaining Assessment

The immunostaining assessment was performed by measuring the fraction by percentage (%) of means the HIF-1*α*, p53, BNIP3, Bcl-2, IAP-2, GLUT1, and Bax staining area in AMB and DF. Images collected from five random fields containing parenchyma and stroma obtained from each sample and acquired using an AxioScope microscope (Carl Zeiss, Oberkochen, DEU) equipped with an AxioCam HRC color camera (Carl Zeiss®). The images were acquired with a magnification of 400x. Diaminobenzidine-stained areas were separated and segmented. Then, they were analysed using immunohistochemistry (IHC) Image Analysis Toolbox of ImageJ Public domain software, developed by Wayne Rasband (NIMH, NIH, Bethesda, MD, EUA, http://rsbweb.nih.gov/ij/) [14, 15]. After image segmentation, the percentage of tumour parenchyma-stained area was measured. Qualitative description of the location of the immunostaining in the plasma membrane, cytoplasm, and nucleus was carried out.

### 2.4. Cell Cultivation

The cell line, AME-1, was grown in culture flasks containing Dulbecco's Modified Eagle Medium F-12 (DMEM/F-12, Gibco, Carlsbad, CA, USA), supplemented with 10% FBS (Gibco®), 10% penicillin and streptomycin (Gibco®), and 1% Fungizone (Gibco®). The cells were kept in an incubator at a temperature of 37°C and a humid atmosphere containing 5% CO2. Cell proliferation was observed daily under an inverted phase contrast microscope (Axiovert 40 C-Zeiss, Jena, TH, DEU), with coupled camera (AxioCam MRc–Zeiss®).

### 2.5. Gene Expression and Network Interaction Analysis

The whole-transcriptome sequencing was performed by Ion Proton platform, as described by De Souza Cruz et al., 2021 [16]. The *Homo sapiens* genome sequence GRCh38.p4 was used to align the reads through Torrent Mapping Alignment Program (TMAP) (https://github.com/iontorrent/TS/tree/master/Analysis/TMAP) to generate a BAM file to be used in the analysis of differential gene expression through GFOLD [17], to AME-1 to generate a list of hypo- or hyperexpressed genes. The network interaction of genes was obtained through the String Database [18] looking for HIF-1alfa gene, with interaction score of 0.7.

### 2.6. Statistical Analysis

To calculate the adherence to the normality curve, the Shapiro–Wilk test was used. The Student's *t*-test was used to assess differences between AMB and DF for samples with normal distribution, and the Mann–Whitney test was used for samples with abnormal distribution (BioEstat®, version 5.3, Instituto Mamiraúa, PA, BR and GraphPad Software Inc., San Diego, CA, USA).

The Poisson regression was used to verify whether there was an association between the expression of HIF-1*α* with p53, GLUT1, Bax, Bcl-2, IAP-2, and BNIP3 proteins. Those variables that presented *p* < 0.05 in the univariate model were included in the multivariate regression model. For all analyses, we adopted a significance level of 5% (STATA 13.0 program, Texas, USA).

Spearman's correlation test was used to verify whether there was a correlation between the expression of the evaluated proteins (BioEstat®, version 5.3, Instituto Mamiraúa, PA, BR).

## 3. Results

### 3.1. AMB and DF Expressed Quantitative Differences for HIF-1*α*, p53, GLUT1, Bax, BNIP3, IAP-2, and Bcl-2

**Figure 1:**
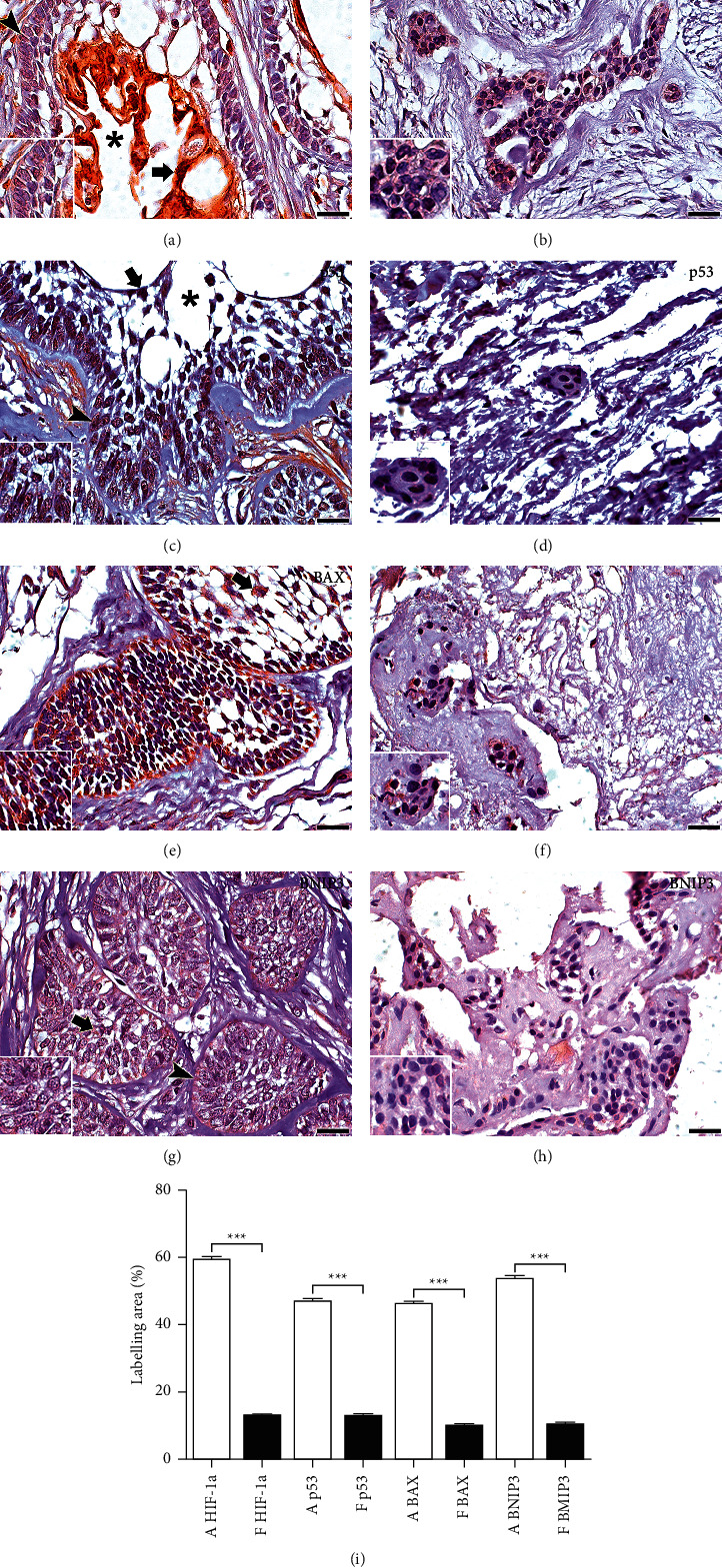
Immunomarking for HIF-1*α*, p53, Bax, and BNIP3 in AMB and FP. HIF-1*α* staining in AMB (a) was nuclear in the solid tumour area (arrowheads) and cytoplasmic and nuclear in cells near the cystic areas of the epithelial islets (arrow and cystic lumen,^*∗*^). p53 immunostaining in AMB (c) is predominant in the nucleus and cytoplasm of cells close to the cystic areas (cystic lumen,^*∗*^) of epithelial islands (arrow). Staining is also detected in selected cells from the basal layer (c) (arrowhead). Bax immunostaining in AMB (e) is predominant in the cytoplasm of cells close to the cystic areas of epithelial islands (arrow). BNIP3 immunostaining in AMB (g) is predominant in the cytoplasm of cells close to the cystic areas of epithelial islands. Marking is also detected in selected cells from the basal and central layers (arrowhead and arrow, resp.). The FP shows a weaker labelling of the HIF-1*α*, p53, Bax, and BNIP3 proteins compared with AMB (b, d, f, h). Scale 100 and 20 *μ*m. Comparison of immunoexpression of the p53, Bax, and BNIP3) and HIF-1*α* in the ameloblastoma (a) and the follicle dental (f). ^*∗∗∗*^*p* < 0.001 (m).

**Figure 2:**
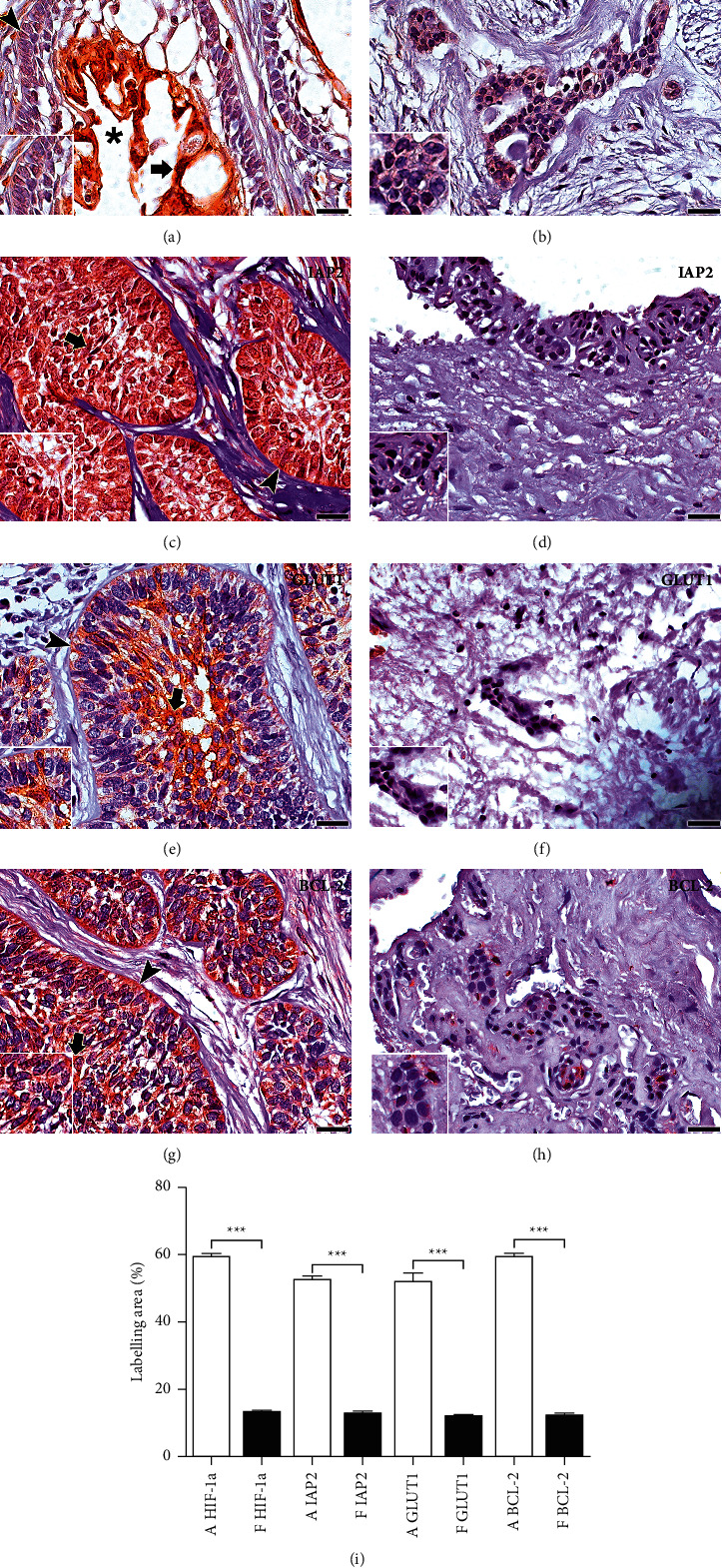
Immunomarking for HIF-1*α*, IAP-2, GLUT1, and Bcl-2 in AMB and FP. HIF-1*α* staining in AMB (a) was nuclear in the solid tumour area (arrowheads) and cytoplasmic and nuclear in cells near the cystic areas of the epithelial islets (arrow and cystic lumen, ^*∗*^). IAP-2 labelling on AMB (c) was predominant in the cytoplasm and nucleus (arrow) of the cells of the epithelial islands. Marking is also detected in selected cells from the basal and central layers (arrowhead and arrow, resp.). GLUT1 immunostaining in AMB (e) is predominant in the cytoplasm of the cells of the basal and central layer of the epithelial islands (arrowhead and arrow, resp.). Bcl-2 immunostaining in AMB (g) is predominant in the cytoplasm of epithelial island cells (arrow). Marking is also detected in selected cells from the basal and central layers (arrowhead and arrow, resp.). The FP shows a weaker labelling of the proteins IAP-2, GLUT1, and Bcl-2 compared with AMB (b, d, f, h). Scale 100 and 20 *μ*m. Comparison of immunoexpression of the HIF-1*α*, IAP-2, GLUT1 and Bcl-2, and HIF-1*α* in the ameloblastoma (a) and the follicle dental (f). ^*∗∗∗*^*p* < 0.001 (m).

HIF-1*α*, p53, GLUT1, Bax, BNIP3, IAP-2, and Bcl-2 proteins showed significantly greater immunoexpression in AMB compared with DF (Table 1; Figures 1 and 2).

### 3.2. Variations in the Expression of HIF-1*α*, p53, GLUT1, Bax, BNIP3, IAP-2, and Bcl-2 in Compartments of Neoplastic Cells

HIF-1*α* was expressed in the nucleus of neoplastic cells in solid areas of the AMB, whereas, in the cystic areas, it was in both the cytoplasm and nucleus of neoplastic cells that lined cystic cavities. The immunoexpression of IAP-2, Bcl-2, and GLUT1 in the peripheral cells of the solid area of the tumour was commonly observed. The immunoexpression of p53, Bax, and BNIP3 was predominantly in the cells of the basal and central layers. Some proteins were predominantly observed in the nucleus, such as p53 and IAP-2, the latter with intense expression in the nucleus, cytoplasm, and plasma membrane. The other proteins mainly showed cytoplasmic expression (Bax, BNIP3, Bcl-2, and GLUT1) (Figures 1 and 2).

### 3.3. Positive Association between HIF-1*α* and GLUT1

The analysis of Poisson regression in a multilevel model was performed with the significant proteins of the univariate analysis (GLUT1, IAP-2, Bcl-2, and BNIP3) in order to determine the influence of HIF-1*α* for each of these proteins. The regression revealed that only GLUT1 (*p* < 0.047) showed a positive association (Table 2).

### 3.4. Positive Correlation between Pro- and Antiapoptotic Proteins

In this study, a positive correlation was found between p53 and IAP-2 (*p* < 0.0113) and between p53 and Bcl-2 (*p* < 0.0071). In addition, there was an important correlation between these antiapoptotic proteins Bcl-2 and IAP-2 (*p* < 0.0003) (Table 3).

### 3.5. Central Role of HIF1-*α* by Transcriptome and Gene Interaction Network

**Figure 3:**
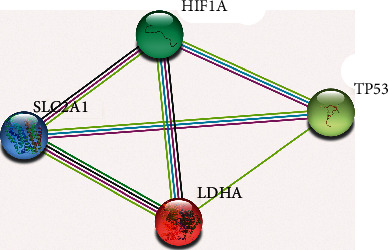
Protein-protein interaction network for HIF-1*α*, GLUT1 (SLC2A1), TP53, and LDHA genes obtained through the STRING platform. It is noteworthy that all interactions observed between proteins (edges connecting the nodes) were experimentally characterized (pink line) and obtained from cured databases (light blue line). The other interactions observed: HIF-1*α* and GLUT1 (SLC2A1); HIF-1*α* and TP53; HIF-1 and LDHA; GLUT1 (SLC2A1) and TP53; GLUT1 (SLC2A1) and LDHA; LDHA and TP53 presented confirmations based on analysis of text mining (yellow line) and coexpression (black line) observed computationally from transcript-transcript interactions.

The gene interaction network generated showed a direct association of HIF-1*α* with GLUT1 (SLC2A1), TP53, and LDHA (Figure 3). Interestingly, GLUT1 also exhibited direct interaction with TP53 and LDHA in the String, validated by experimental studies.

The transcriptomic analysis showed significant expression of HIF-1*α* (495 reads), IAP2 and BNIP3 (72 reads), BCL2 (60 reads), and GLUT1 (56 reads) in the AME-1 samples.

## 4. Discussion

The studied proteins were significantly more expressed in AMB compared with DF. Additionally, a positive association was observed between HIF-1*α* and GLUT1. HIF-1*α* was expressed mainly in the nucleus of cells that made up the solid areas of the tumour and the nucleus and cytoplasm of the neoplastic epithelial cells that line cystic areas. Immunoexpression of antiapoptotic proteins has been commonly observed in peripheral columnar cells of solid areas of the tumour. In turn, the expression of proapoptotic proteins was predominant in cells of the basal and central layers of the neoplastic islands.

Cells proliferate during the tumour process and access to oxygen in the central area of the tumour begins to be restricted, which leads to hypoxia [8, 19]. Hypoxia or hypoxia gradients occur in most slow-growing solid tumours and can result in pleiotropic effects that contribute significantly to tumour aggressiveness. Hypoxia is also associated with a more aggressive phenotype, which affects angiogenesis and cellular invasiveness [20].

**Figure 4:**
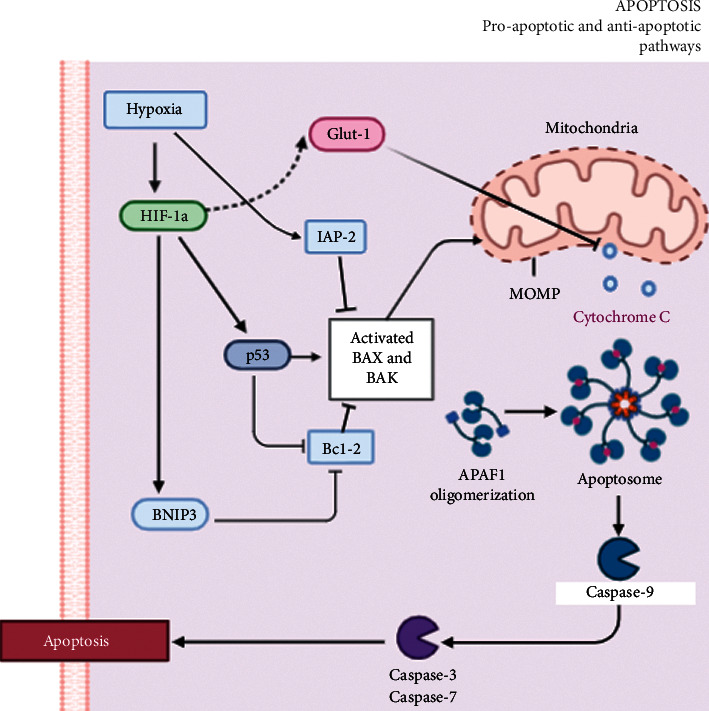
Schematic representation of hypoxia-induced proapoptotic and antiapoptotic signalling pathways. The involvement of HIF-1*α* is described in these pathways. The solid lines indicate a direct interaction; the dashed line indicates an indirect interaction. Induction (arrow); inhibition (headless arrow). APAF-1, cytochrome C, and procaspase 9 form the apoptosome that activates caspase 9. APAF-1: apoptotic protease activating factor 1; BNIP3: protein 3 that interacts with BCL-2/adenovirus E1B of 19 kDa; HIF-1*α*: hypoxia-inducible factor 1; IAP-2: inhibitor of apoptosis protein 2; GLUT1: glucose transporter; MOMP: mitochondrial outer membrane permeabilisation; caspase 9 (inducer); caspases 3 (irreversible apoptosis marker); caspase 7 (effector). *By Katherine J. Palma Valladares with*https://app.biorender.com/.

This study suggests possible signalling pathways that could contribute to the development of AMB, where HIF-1*α* is the main inducer of the series of adaptive responses of cells that are subjected to hypoxia stress [6, 19]. When the cell is in an excellent condition, it responds physiologically and shows adequate homeostasis between death and cell survival [3]. This whole process is mediated by proteins such as p53 that are activated by HIF-1*α*. In turn, p53 stimulates proteins such as Bax (protein required for the onset of apoptosis) and also inhibits Bcl-2 (antiapoptotic protein overexpressed in benign and malignant neoplasms) [5]. If Bax is unable to move in to the outer mitochondrial membrane to initiate apoptosis, it is due to the inhibition of IAP-2 that is present in the cell under conditions of severe hypoxia, making it resistant to apoptosis [21] (Figure 4).

HIF-1*α* mediates cell survival and resistance to apoptosis in hypoxia; probably, one of these mechanisms is through alterations in cellular energy metabolism by increasing glucose uptake and glycolysis by GLUT1, and this could confer this resistant phenotype [6, 22]. In addition, if there is any change in the balance offered by proteins such as p53, for example a mutation, this phenomenon would favour the function of antiapoptotic proteins and, therefore, tumour growth [23].

During hypoxia, the alpha subunit stabilises, translocates, and accumulates in the nucleus with the beta subunit (HIF-*β*), where it will bind to specific DNA sequences, activating genes involved in adaptation to hypoxia, cell survival, angiogenesis, and metastasis [10–12]. Therefore, if HIF-1*α* still stabilises in the cytoplasm in some way, this heterodimerisation possibly does not occur in the nucleus, which would facilitate the apoptosis process to take place, as observed in the immunoexpression of HIF-1*α* in the epithelial cells near the cystic areas. The opposite occurs in cells that showed nuclear immunoexpression, which suggests resistance to apoptosis.

The expression of antiapoptotic proteins in the basal layer of AMB cells may suggest not only a proliferative activity, but also the inhibition of cell death, characteristics that reflect the growth potential of this neoplasm [24].

There is an evident difference between the immunoexpression of AMB and DF (nonpathological odontogenic epithelium), suggesting the proapoptotic or antiapoptotic intervention of HIF-1*α*, p53, GLUT1, Bax, BNIP3, IAP-2, and Bcl-2 in AMB pathological neoplastic processes.

HIF-1*α* plays an important role in the protection of solid tumours against hypoxia, preventing apoptosis or increasing anaerobic metabolism [5, 6, 25]. This includes processes that lead to better oxygen supply (angiogenesis), increased glycolytic metabolism, and a shift from oxidative phosphorylation to anaerobic glycolysis [26].

This study has shown that GLUT1 has a strong association with HIF-1*α*. Semenza et al. hypothesize that increased glycolytic metabolism could confer this resistant phenotype. They therefore examined the expression levels of GLUT1, one of the key glycolytic enzymes, which is known to be the inducible target gene of HIF-1*α* [6]. Glucose inhibits the translocation of cytochrome C from the mitochondria to the cytosol, an event that is necessary to trigger apoptosis [26]. GLUT1 is not considered an antiapoptotic protein as such; however, under certain conditions, the positive regulation of GLUT1 mediated by HIF-1*α* in hypoxia appears to confer resistance to apoptosis [20, 26], an effect that supports the results obtained.

DNA fragmentation is an important event in the elimination of the genome of apoptotic cells and subsequent cell death. Therefore, intense nuclear expression of p53 indicates the beginning of apoptosis [6]. In this study, the p53 predominantly nuclear expression in AMB may suggest that the localisation favours apoptosis. IAP-2 also showed intense nuclear expression, possibly pointing to increased resistance to apoptosis [6].

This study demonstrates an important association between p53 and IAP-2 and between p53 and Bcl-2, with an overexpression of p53 (proapoptotic) and the increased expression of Bcl-2 and IAP-2 (antiapoptotics). The overexpression of p53 in conventional AMB and malignant AMB may reflect a mutational p53 protein that plays an oncogenic role promoting tumour growth [25]. The p53 mutation leads to promotion of the function of antiapoptotic proteins. Therefore, the importance of cell cycle aberration and uncontrolled proliferation resulting from the p53 mutation is discussed [27].

Bcl-2 is a protein that is overexpressed in benign and malignant neoplasms; the overexpression of Bcl-2 positively modulates proteins such as IAP-2, inhibiting apoptosis [28]. In this study, we found a positive correlation between Bcl-2 and IAP-2 proteins. The latter is independent of HIF-1*α* but is generally expressed under conditions of severe hypoxia [29]. This association, together with a possible p53 mutation, could suggest an explanation for the aggressive behaviour of AMB.

Data from gene expression analysis by RNA-Seq, gene interaction network, and immunohistochemical study demonstrate the central role of HIF-1*α* in relation to direct activation of GLUT1 (SLC2A1), TP53, and LDHA genes under hypoxia, the activation of anaerobic metabolism, and the avoidance of apoptosis in previous studies [5, 6, 22, 25].

However, the direct correlation between HIF-1*α* with TP53 and LDHA has not yet been described in any study. The LDHA gene expresses an enzyme that mediates metabolic plasticity in the bidirectional conversion of pyruvate and lactate, which favours the invasiveness and progression of oral squamous cell carcinoma [30, 31].

It is important to emphasize that alterations in cell metabolism can lead to critical states due to interrupted or increased events such as apoptosis, as shown in this study, favoring tumour growth and/or bone resorption, respectively. Hypoxia has been documented to be an important phenomenon that also promotes osteogenesis and osteogenic differentiation of stem cells [32, 33]. In this way, a type of inductive therapy could be implemented through natural or present biomolecules in the organism that promote the stimulation or enhancement of these regenerative events, as suggested by recent studies [33, 34]; in the future, this could counteract the high probability of recurrence of this pathology.

It is worth highlighting the need for further studies, such as mechanistic assays, which can suppress the expression of HIF-1 alpha and see the influence of this lock on cell apoptosis, given the limitations that the immunohistochemical study may have.

## 5. Conclusion

Therefore, the observed associations indicate that hypoxia is possibly related to antiapoptotic events. This suggests an important role for GLUT1 (dependent on HIF-1*α*), Bcl-2, and IAP-2 stimulated by the overexpression of p53 and its possible mutation, which suggests a resistance to apoptosis, favouring cell survival, growth, and probably the aggressiveness of AMB. These proteins may be important targets for better understanding the tumorigenesis and treatment of AMB.

## Figures and Tables

**Table 1:** The *p* values of HIF-1*α*, p53, BNIP3, Bcl-2, IAP-2, GLUT1, and Bax with expression in AMB and DF, Student's *t*, and Mann–Whitney tests.

Protein	AME (*n* = 20)		DF (*n* = 10)		*p* value
	Mean	SD	Mean	SD	
HIF-1*α*	59.04	±5.61	12.81	±1.68	<0.0001
p53	46.26	±4.24	12.68	±2.61	<0.0001
BNIP3	53.31	±6.11	10.22	±1.57	<0.0001
Bcl-2	59.03	±5.64	11.87	±3.14	<0.0001
IAP-2	52.21	±6.02	11.94	±1.72	<0.0001
GLUT1	51.65	±11.19	11.71	±1.40	<0.0001
Bax	45.94	±5.05	9.81	±1.67	<0.0001

AMB: ameloblastoma; DF: dental follicle.

**Table 2:** Univariate Poisson regression analysis (unadjusted RR) between p53, GLUT1, Bax, Bcl-2, IAP-2, and BNIP3 with HIF-1*α* and multiple analysis (adjusted RR) between GLUT1, Bcl-2, IAP-2, and BNIP3.

Dependent	Protein	Unadjusted RR	*p* value	Adjusted RR	*p* value	95% CI	
HIF-1*α*	p53	1.006101	0.188				
	GLUT1	1.004928	0.001	1.002975	0.047	1.000033	1.005926
	Bax	1.001577	0.573				
	Bcl-2	1.007792	0.009	1.003057	0.468	0.9948193	1.011362
	IAP-2	1.007991	<0.001	1.002849	0.355	0.9968161	1.008918
	BNIP3	1.006999	0.007	1.003297	0.280	0.9973217	1.009309

RR: rate ratio; 95% CI: 95% confidence interval.

**Table 3:** Spearman correlation test positive in the area of protein labelling in ameloblastoma.

Protein 1	Protein 2	*r* ^2^	*p* value
p53	Bcl-2	0.5817	0.0071
	IAP-2	0.5534	0.0113
Bcl-2	IAP-2	0.7248	0.0003

*r* ^2^: Spearman's correlation coefficient.

## Data Availability

The datasets generated during and/or analysed during the current study are available from the corresponding author on reasonable request.
